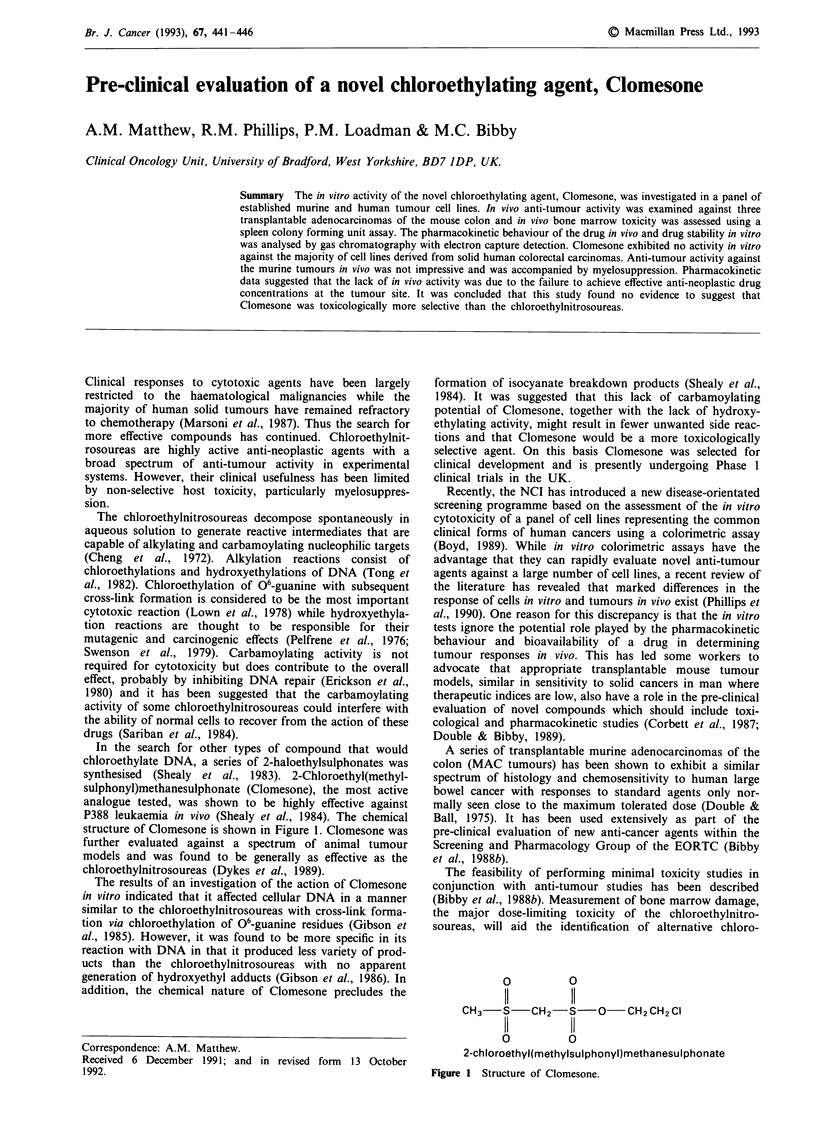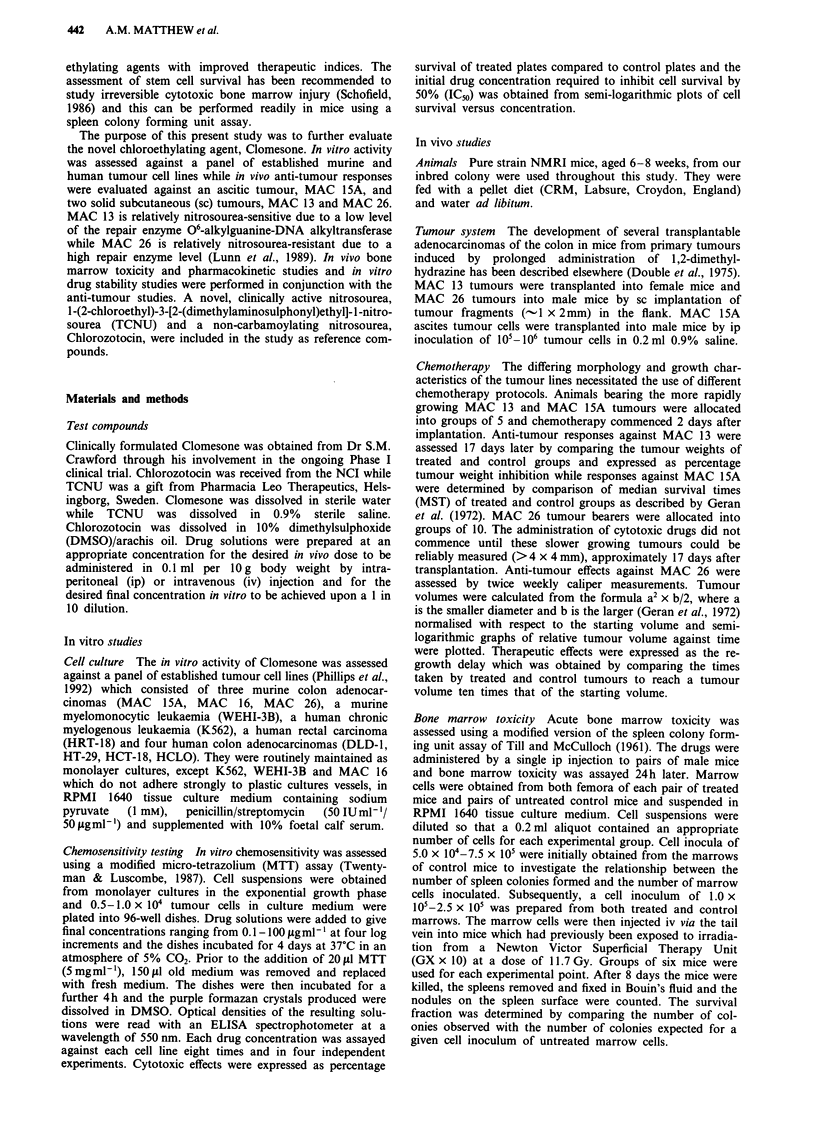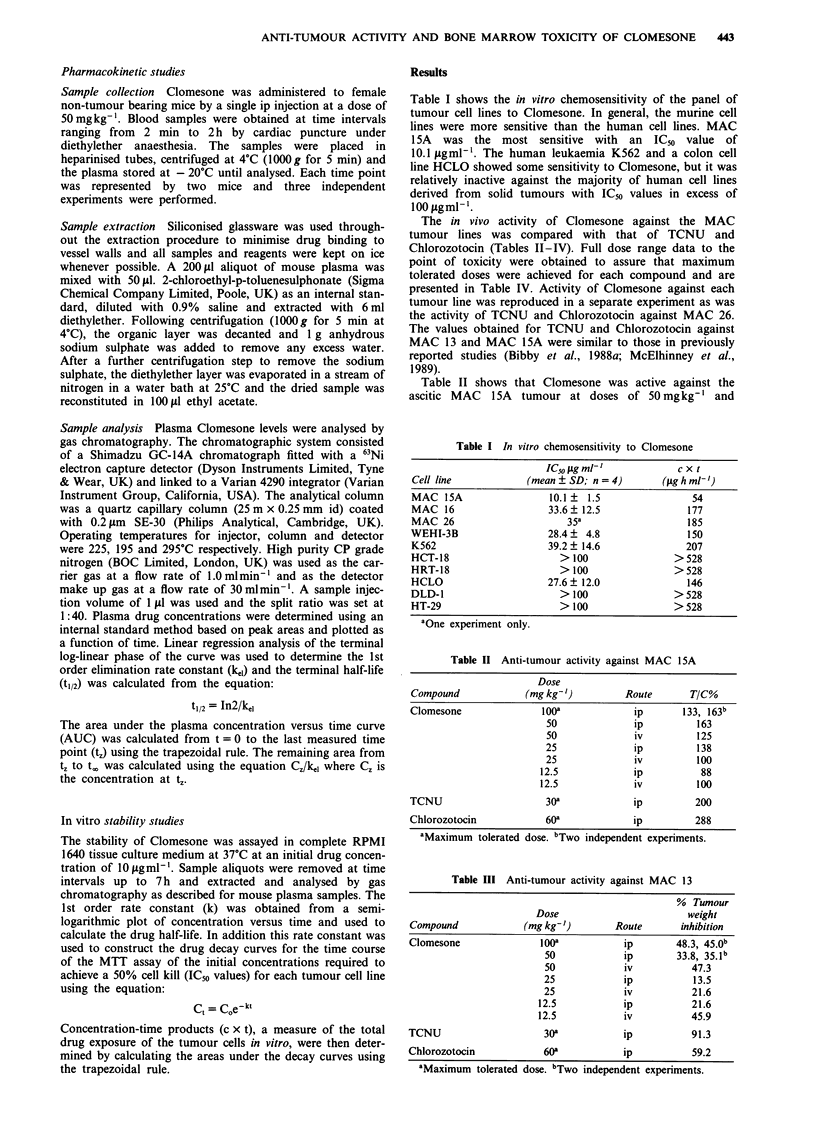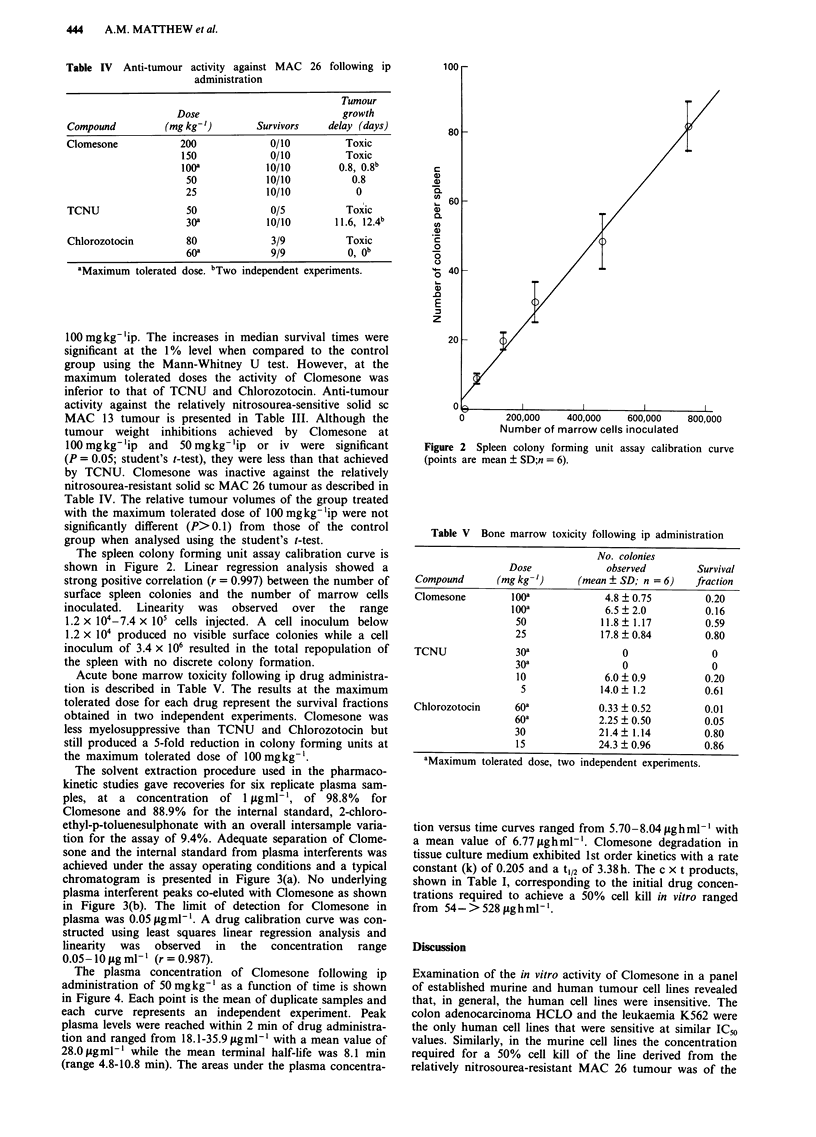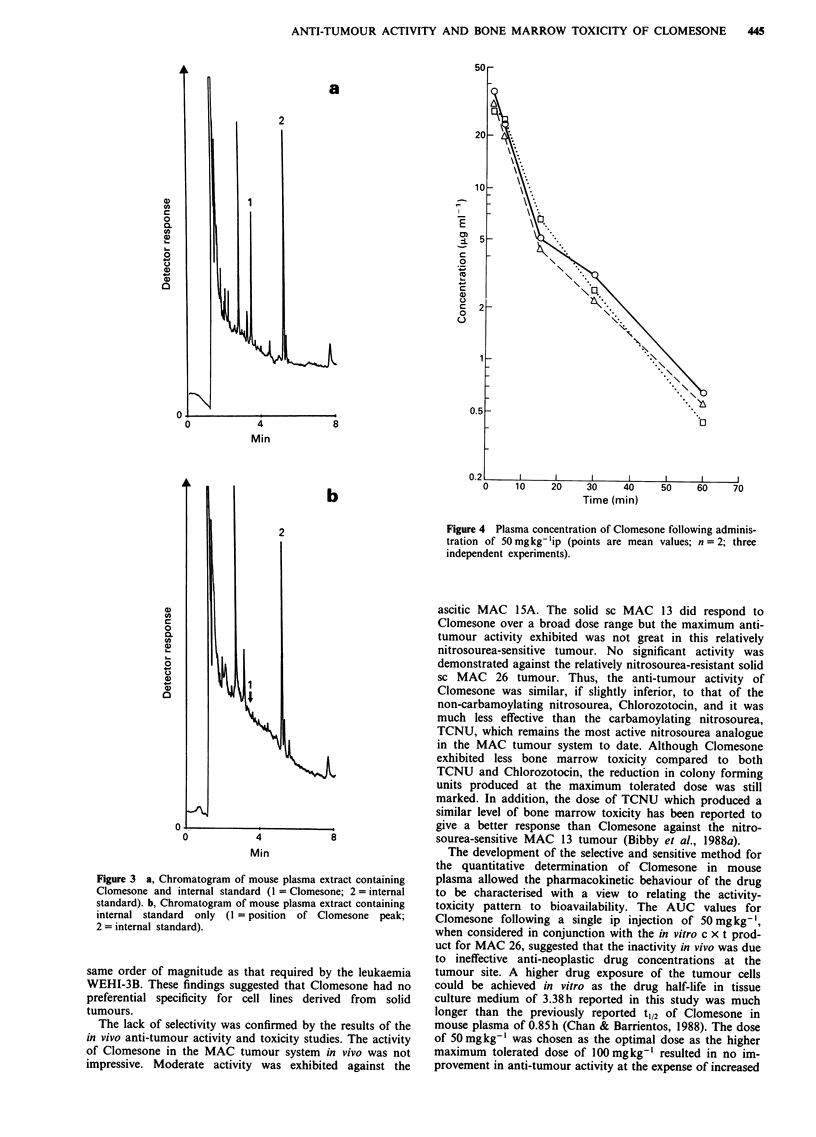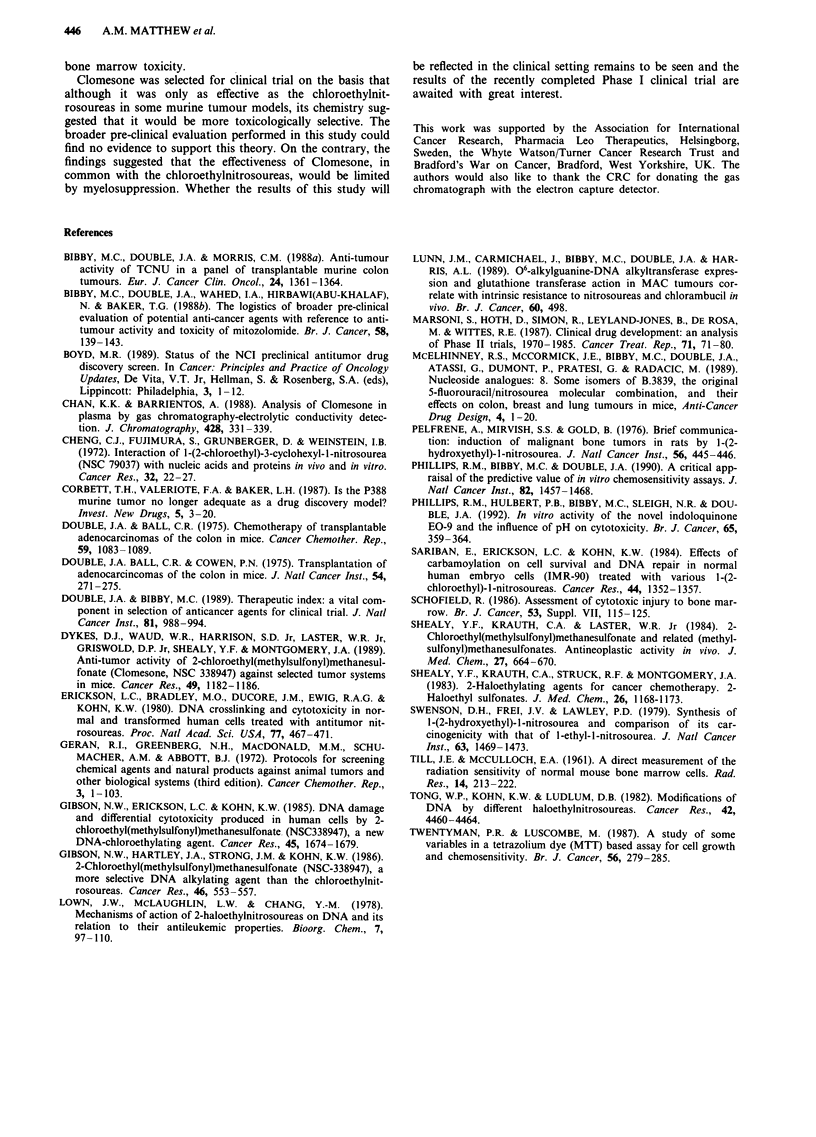# Pre-clinical evaluation of a novel chloroethylating agent, Clomesone.

**DOI:** 10.1038/bjc.1993.85

**Published:** 1993-03

**Authors:** A. M. Matthew, R. M. Phillips, P. M. Loadman, M. C. Bibby

**Affiliations:** Clinical Oncology Unit, University of Bradford, West Yorkshire, UK.

## Abstract

The in vitro activity of the novel chloroethylating agent, Clomesone, was investigated in a panel of established murine and human tumour cell lines. In vivo anti-tumour activity was examined against three transplantable adenocarcinomas of the mouse colon and in vivo bone marrow toxicity was assessed using a spleen colony forming unit assay. The pharmacokinetic behaviour of the drug in vivo and drug stability in vitro was analysed by gas chromatography with electron capture detection. Clomesone exhibited no activity in vitro against the majority of cell lines derived from solid human colorectal carcinomas. Anti-tumour activity against the murine tumours in vivo was not impressive and was accompanied by myelosuppression. Pharmacokinetic data suggested that the lack of in vivo activity was due to the failure to achieve effective anti-neoplastic drug concentrations at the tumour site. It was concluded that this study found no evidence to suggest that Clomesone was toxicologically more selective than the chloroethylnitrosoureas.


Br. J. Cancer (1993), 67, 441-446 © Macmillan Press Ltd., 1993

### Pre-clinical evaluation of a novel chloroethylating agent, Clomesone

A.M. Matthew, R.M. Phillips, P.M. Loadman & M.C. Bibby

*Clinical Oncology Unit, University of Bradford, West Yorkshire, BD7 1DP, UK.*

**Summary** The *in vitro* activity of the novel chloroethylating agent, Clomesone, was investigated in a panel of established murine and human tumour cell lines. *In vivo* anti-tumour activity was examined against three transplantable adenocarcinomas of the mouse colon and *in vivo* bone marrow toxicity was assessed using a spleen colony forming unit assay. The pharmacokinetic behaviour of the drug *in vivo* and drug stability *in vitro* was analysed by gas chromatography with electron capture detection. Clomesone exhibited no activity *in vitro* against the majority of cell lines derived from solid human colorectal carcinomas. Anti-tumour activity against the murine tumours *in vivo* was not impressive and was accompanied by myelosuppression. Pharmacokinetic data suggested that the lack of *in vivo* activity was due to the failure to achieve effective anti-neoplastic drug concentrations at the tumour site. It was concluded that this study found no evidence to suggest that Clomesone was toxicologically more selective than the chloroethylnitrosoureas.

Clinical responses to cytotoxic agents have been largely restricted to the haematological malignancies while the majority of human solid tumours have remained refractory to chemotherapy (Marsoni *et al.*, 1987). Thus the search for more effective compounds has continued. Chloroethylnitrosoureas are highly active anti-neoplastic agents with a broad spectrum of anti-tumour activity in experimental systems. However, their clinical usefulness has been limited by non-selective host toxicity, particularly myelosuppression.

The chloroethylnitrosoureas decompose spontaneously in aqueous solution to generate reactive intermediates that are capable of alkylating and carbamoylating nucleophilic targets (Cheng *et al.*, 1972). Alkylation reactions consist of chloroethylations and hydroxyethylations of DNA (Tong *et al.*, 1982). Chloroethylation of O$^6$-guanine with subsequent cross-link formation is considered to be the most important cytotoxic reaction (Lown *et al.*, 1978) while hydroxyethylation reactions are thought to be responsible for their mutagenic and carcinogenic effects (Pelfrene *et al.*, 1976; Swenson *et al.*, 1979). Carbamoylating activity is not required for cytotoxicity but does contribute to the overall effect, probably by inhibiting DNA repair (Erickson *et al.*, 1980) and it has been suggested that the carbamoylating activity of some chloroethylnitrosoureas could interfere with the ability of normal cells to recover from the action of these drugs (Sariban *et al.*, 1984).

In the search for other types of compound that would chloroethylate DNA, a series of 2-haloethylsulphonates was synthesised (Shealy *et al.*, 1983). 2-Chloroethyl(methylsulphonyl)methanesulphonate (Clomesone), the most active analogue tested, was shown to be highly effective against P388 leukaemia *in vivo* (Shealy *et al.*, 1984). The chemical structure of Clomesone is shown in Figure 1. Clomesone was further evaluated against a spectrum of animal tumour models and was found to be generally as effective as the chloroethylnitrosoureas (Dykes *et al.*, 1989).

The results of an investigation of the action of Clomesone *in vitro* indicated that it affected cellular DNA in a manner similar to the chloroethylnitrosoureas with cross-link formation *via* chloroethylation of O$^6$-guanine residues (Gibson *et al.*, 1985). However, it was found to be more specific in its reaction with DNA in that it produced less variety of products than the chloroethylnitrosoureas with no apparent generation of hydroxyethyl adducts (Gibson *et al.*, 1986). In addition, the chemical nature of Clomesone precludes the formation of isocyanate breakdown products (Shealy *et al.*, 1984). It was suggested that this lack of carbamoylating potential of Clomesone, together with the lack of hydroxyethylating activity, might result in fewer unwanted side reactions and that Clomesone would be a more toxicologically selective agent. On this basis Clomesone was selected for clinical development and is presently undergoing Phase 1 clinical trials in the UK.

Recently, the NCI has introduced a new disease-orientated screening programme based on the assessment of the *in vitro* cytotoxicity of a panel of cell lines representing the common clinical forms of human cancers using a colorimetric assay (Boyd, 1989). While *in vitro* colorimetric assays have the advantage that they can rapidly evaluate novel anti-tumour agents against a large number of cell lines, a recent review of the literature has revealed that marked differences in the response of cells *in vitro* and tumours *in vivo* exist (Phillips *et al.*, 1990). One reason for this discrepancy is that the *in vitro* tests ignore the potential role played by the pharmacokinetic behaviour and bioavailability of a drug in determining tumour responses *in vivo*. This has led some workers to advocate that appropriate transplantable mouse tumour models, similar in sensitivity to solid cancers in man where therapeutic indices are low, also have a role in the pre-clinical evaluation of novel compounds which should include toxicological and pharmacokinetic studies (Corbett *et al.*, 1987; Double & Bibby, 1989).

A series of transplantable murine adenocarcinomas of the colon (MAC tumours) has been shown to exhibit a similar spectrum of histology and chemosensitivity to human large bowel cancer with responses to standard agents only normally seen close to the maximum tolerated dose (Double & Ball, 1975). It has been used extensively as part of the pre-clinical evaluation of new anti-cancer agents within the Screening and Pharmacology Group of the EORTC (Bibby *et al.*, 1988*b*).

The feasibility of performing minimal toxicity studies in conjunction with anti-tumour studies has been described (Bibby *et al.*, 1988*b*). Measurement of bone marrow damage, the major dose-limiting toxicity of the chloroethylnitrosoureas, will aid the identification of alternative chloro-

2-chloroethyl(methylsulphonyl)methanesulphonate

**Figure 1** Structure of Clomesone.

Correspondence: A.M. Matthew.
Received 6 December 1991; and in revised form 13 October 1992.

ethylating agents with improved therapeutic indices. The assessment of stem cell survival has been recommended to study irreversible cytotoxic bone marrow injury (Schofield, 1986) and this can be performed readily in mice using a spleen colony forming unit assay.

The purpose of this present study was to further evaluate the novel chloroethylating agent, Clomesone. *In vitro* activity was assessed against a panel of established murine and human tumour cell lines while *in vivo* anti-tumour responses were evaluated against an ascitic tumour, MAC 15A, and two solid subcutaneous (sc) tumours, MAC 13 and MAC 26. MAC 13 is relatively nitrosourea-sensitive due to a low level of the repair enzyme $O^6$-alkylguanine-DNA alkyltransferase while MAC 26 is relatively nitrosourea-resistant due to a high repair enzyme level (Lunn et al., 1989). *In vivo* bone marrow toxicity and pharmacokinetic studies and *in vitro* drug stability studies were performed in conjunction with the anti-tumour studies. A novel, clinically active nitrosourea, 1-(2-chloroethyl)-3-[2-(dimethylaminosulphonyl)ethyl]-1-nitrosourea (TCNU) and a non-carbamoylating nitrosourea, Chlorozotocin, were included in the study as reference compounds.

### Materials and methods

### *Test compounds*

Clinically formulated Clomesone was obtained from Dr S.M. Crawford through his involvement in the ongoing Phase I clinical trial. Chlorozotocin was received from the NCI while TCNU was a gift from Pharmacia Leo Therapeutics, Helsingborg, Sweden. Clomesone was dissolved in sterile water while TCNU was dissolved in 0.9% sterile saline. Chlorozotocin was dissolved in 10% dimethylsulphoxide (DMSO)/arachis oil. Drug solutions were prepared at an appropriate concentration for the desired *in vivo* dose to be administered in 0.1 ml per 10 g body weight by intraperitoneal (ip) or intravenous (iv) injection and for the desired final concentration *in vitro* to be achieved upon a 1 in 10 dilution.

### In vitro *studies*

*Cell culture* The *in vitro* activity of Clomesone was assessed against a panel of established tumour cell lines (Phillips et al., 1992) which consisted of three murine colon adenocarcinomas (MAC 15A, MAC 16, MAC 26), a murine myelomonocytic leukaemia (WEHI-3B), a human chronic myelogenous leukaemia (K562), a human rectal carcinoma (HRT-18) and four human colon adenocarcinomas (DLD-1, HT-29, HCT-18, HCLO). They were routinely maintained as monolayer cultures, except K562, WEHI-3B and MAC 16 which do not adhere strongly to plastic cultures vessels, in RPMI 1640 tissue culture medium containing sodium pyruvate (1 mM), penicillin/streptomycin ($50\,IU\,ml^{-1}$/$50\,\mu g\,ml^{-1}$) and supplemented with 10% foetal calf serum.

*Chemosensitivity testing* *In vitro* chemosensitivity was assessed using a modified micro-tetrazolium (MTT) assay (Twentyman & Luscombe, 1987). Cell suspensions were obtained from monolayer cultures in the exponential growth phase and $0.5-1.0 \times 10^4$ tumour cells in culture medium were plated into 96-well dishes. Drug solutions were added to give final concentrations ranging from $0.1-100\,\mu g\,ml^{-1}$ at four log increments and the dishes incubated for 4 days at 37°C in an atmosphere of 5% $CO_2$. Prior to the addition of 20 µl MTT ($5\,mg\,ml^{-1}$), 150 µl old medium was removed and replaced with fresh medium. The dishes were then incubated for a further 4 h and the purple formazan crystals produced were dissolved in DMSO. Optical densities of the resulting solutions were read with an ELISA spectrophotometer at a wavelength of 550 nm. Each drug concentration was assayed against each cell line eight times and in four independent experiments. Cytotoxic effects were expressed as percentage survival of treated plates compared to control plates and the initial drug concentration required to inhibit cell survival by 50% ($IC_{50}$) was obtained from semi-logarithmic plots of cell survival versus concentration.

### In vivo *studies*

*Animals* Pure strain NMRI mice, aged 6–8 weeks, from our inbred colony were used throughout this study. They were fed with a pellet diet (CRM, Labsure, Croydon, England) and water *ad libitum*.

*Tumour system* The development of several transplantable adenocarcinomas of the colon in mice from primary tumours induced by prolonged administration of 1,2-dimethylhydrazine has been described elsewhere (Double et al., 1975). MAC 13 tumours were transplanted into female mice and MAC 26 tumours into male mice by sc implantation of tumour fragments ($\sim 1 \times 2$ mm) in the flank. MAC 15A ascites tumour cells were transplanted into male mice by ip inoculation of $10^5-10^6$ tumour cells in 0.2 ml 0.9% saline.

*Chemotherapy* The differing morphology and growth characteristics of the tumour lines necessitated the use of different chemotherapy protocols. Animals bearing the more rapidly growing MAC 13 and MAC 15A tumours were allocated into groups of 5 and chemotherapy commenced 2 days after implantation. Anti-tumour responses against MAC 13 were assessed 17 days later by comparing the tumour weights of treated and control groups and expressed as percentage tumour weight inhibition while responses against MAC 15A were determined by comparison of median survival times (MST) of treated and control groups as described by Geran et al. (1972). MAC 26 tumour bearers were allocated into groups of 10. The administration of cytotoxic drugs did not commence until these slower growing tumours could be reliably measured ($>4 \times 4$ mm), approximately 17 days after transplantation. Anti-tumour effects against MAC 26 were assessed by twice weekly caliper measurements. Tumour volumes were calculated from the formula $a^2 \times b/2$, where a is the smaller diameter and b is the larger (Geran et al., 1972) normalised with respect to the starting volume and semi-logarithmic graphs of relative tumour volume against time were plotted. Therapeutic effects were expressed as the re-growth delay which was obtained by comparing the times taken by treated and control tumours to reach a tumour volume ten times that of the starting volume.

*Bone marrow toxicity* Acute bone marrow toxicity was assessed using a modified version of the spleen colony forming unit assay of Till and McCulloch (1961). The drugs were administered by a single ip injection to pairs of male mice and bone marrow toxicity was assayed 24 h later. Marrow cells were obtained from both femora of each pair of treated mice and pairs of untreated control mice and suspended in RPMI 1640 tissue culture medium. Cell suspensions were diluted so that a 0.2 ml aliquot contained an appropriate number of cells for each experimental group. Cell inocula of $5.0 \times 10^4-7.5 \times 10^5$ were initially obtained from the marrows of control mice to investigate the relationship between the number of spleen colonies formed and the number of marrow cells inoculated. Subsequently, a cell inoculum of $1.0 \times 10^5-2.5 \times 10^5$ was prepared from both treated and control marrows. The marrow cells were then injected iv *via* the tail vein into mice which had previously been exposed to irradiation from a Newton Victor Superficial Therapy Unit (GX × 10) at a dose of 11.7 Gy. Groups of six mice were used for each experimental point. After 8 days the mice were killed, the spleens removed and fixed in Bouin's fluid and the nodules on the spleen surface were counted. The survival fraction was determined by comparing the number of colonies observed with the number of colonies expected for a given cell inoculum of untreated marrow cells.

*Pharmacokinetic studies*

*Sample collection* Clomesone was administered to female non-tumour bearing mice by a single ip injection at a dose of 50 mg kg$^{-1}$. Blood samples were obtained at time intervals ranging from 2 min to 2 h by cardiac puncture under diethylether anaesthesia. The samples were placed in heparinised tubes, centrifuged at 4°C (1000 *g* for 5 min) and the plasma stored at − 20°C until analysed. Each time point was represented by two mice and three independent experiments were performed.

*Sample extraction* Siliconised glassware was used throughout the extraction procedure to minimise drug binding to vessel walls and all samples and reagents were kept on ice whenever possible. A 200 μl aliquot of mouse plasma was mixed with 50 μl. 2-chloroethyl-p-toluenesulphonate (Sigma Chemical Company Limited, Poole, UK) as an internal standard, diluted with 0.9% saline and extracted with 6 ml diethylether. Following centrifugation (1000 *g* for 5 min at 4°C), the organic layer was decanted and 1 g anhydrous sodium sulphate was added to remove any excess water. After a further centrifugation step to remove the sodium sulphate, the diethylether layer was evaporated in a stream of nitrogen in a water bath at 25°C and the dried sample was reconstituted in 100 μl ethyl acetate.

*Sample analysis* Plasma Clomesone levels were analysed by gas chromatography. The chromatographic system consisted of a Shimadzu GC-14A chromatograph fitted with a $^{63}$Ni electron capture detector (Dyson Instruments Limited, Tyne & Wear, UK) and linked to a Varian 4290 integrator (Varian Instrument Group, California, USA). The analytical column was a quartz capillary column (25 m × 0.25 mm id) coated with 0.2 μm SE-30 (Philips Analytical, Cambridge, UK). Operating temperatures for injector, column and detector were 225, 195 and 295°C respectively. High purity CP grade nitrogen (BOC Limited, London, UK) was used as the carrier gas at a flow rate of 1.0 ml min$^{-1}$ and as the detector make up gas at a flow rate of 30 ml min$^{-1}$. A sample injection volume of 1 μl was used and the split ratio was set at 1:40. Plasma drug concentrations were determined using an internal standard method based on peak areas and plotted as a function of time. Linear regression analysis of the terminal log-linear phase of the curve was used to determine the 1st order elimination rate constant ($k_{el}$) and the terminal half-life ($t_{1/2}$) was calculated from the equation:

$$t_{1/2} = \ln 2/k_{el}$$

The area under the plasma concentration versus time curve (AUC) was calculated from t = 0 to the last measured time point ($t_z$) using the trapezoidal rule. The remaining area from $t_z$ to $t_\infty$ was calculated using the equation $C_z/k_{el}$ where $C_z$ is the concentration at $t_z$.

In vitro *stability studies*

The stability of Clomesone was assayed in complete RPMI 1640 tissue culture medium at 37°C at an initial drug concentration of 10 μg ml$^{-1}$. Sample aliquots were removed at time intervals up to 7 h and extracted and analysed by gas chromatography as described for mouse plasma samples. The 1st order rate constant (k) was obtained from a semi-logarithmic plot of concentration versus time and used to calculate the drug half-life. In addition this rate constant was used to construct the drug decay curves for the time course of the MTT assay of the initial concentrations required to achieve a 50% cell kill ($IC_{50}$ values) for each tumour cell line using the equation:

$$C_t = C_0 e^{-kt}$$

Concentration-time products (c × t), a measure of the total drug exposure of the tumour cells *in vitro*, were then determined by calculating the areas under the decay curves using the trapezoidal rule.

**Results**

Table I shows the *in vitro* chemosensitivity of the panel of tumour cell lines to Clomesone. In general, the murine cell lines were more sensitive than the human cell lines. MAC 15A was the most sensitive with an $IC_{50}$ value of 10.1 μg ml$^{-1}$. The human leukaemia K562 and a colon cell line HCLO showed some sensitivity to Clomesone, but it was relatively inactive against the majority of human cell lines derived from solid tumours with $IC_{50}$ values in excess of 100 μg ml$^{-1}$.

The *in vivo* activity of Clomesone against the MAC tumour lines was compared with that of TCNU and Chlorozotocin (Tables II–IV). Full dose range data to the point of toxicity were obtained to assure that maximum tolerated doses were achieved for each compound and are presented in Table IV. Activity of Clomesone against each tumour line was reproduced in a separate experiment as was the activity of TCNU and Chlorozotocin against MAC 26. The values obtained for TCNU and Chlorozotocin against MAC 13 and MAC 15A were similar to those in previously reported studies (Bibby *et al.*, 1988*a*; McElhinney *et al.*, 1989).

Table II shows that Clomesone was active against the ascitic MAC 15A tumour at doses of 50 mg kg$^{-1}$ and

**Table I** *In vitro* chemosensitivity to Clomesone

| *Cell line* | *$IC_{50}$ μg ml$^{-1}$ (mean ± SD; n = 4)* | *c × t (μg h ml$^{-1}$)* |
|---|---|---|
| MAC 15A | 10.1 ± 1.5 | 54 |
| MAC 16 | 33.6 ± 12.5 | 177 |
| MAC 26 | 35[a] | 185 |
| WEHI-3B | 28.4 ± 4.8 | 150 |
| K562 | 39.2 ± 14.6 | 207 |
| HCT-18 | > 100 | > 528 |
| HRT-18 | > 100 | > 528 |
| HCLO | 27.6 ± 12.0 | 146 |
| DLD-1 | > 100 | > 528 |
| HT-29 | > 100 | > 528 |

[a]One experiment only.

**Table II** Anti-tumour activity against MAC 15A

| *Compound* | *Dose (mg kg$^{-1}$)* | *Route* | *T/C%* |
|---|---|---|---|
| Clomesone | 100[a] | ip | 133, 163[b] |
| | 50 | ip | 163 |
| | 50 | iv | 125 |
| | 25 | ip | 138 |
| | 25 | iv | 100 |
| | 12.5 | ip | 88 |
| | 12.5 | iv | 100 |
| TCNU | 30[a] | ip | 200 |
| Chlorozotocin | 60[a] | ip | 288 |

[a]Maximum tolerated dose. [b]Two independent experiments.

**Table III** Anti-tumour activity against MAC 13

| *Compound* | *Dose (mg kg$^{-1}$)* | *Route* | *% Tumour weight inhibition* |
|---|---|---|---|
| Clomesone | 100[a] | ip | 48.3, 45.0[b] |
| | 50 | ip | 33.8, 35.1[b] |
| | 50 | iv | 47.3 |
| | 25 | ip | 13.5 |
| | 25 | iv | 21.6 |
| | 12.5 | ip | 21.6 |
| | 12.5 | iv | 45.9 |
| TCNU | 30[a] | ip | 91.3 |
| Chlorozotocin | 60[a] | ip | 59.2 |

[a]Maximum tolerated dose. [b]Two independent experiments.

**Table IV** Anti-tumour activity against MAC 26 following ip administration

| Compound | Dose ($mg\,kg^{-1}$) | Survivors | Tumour growth delay (days) |
|---|---|---|---|
| Clomesone | 200 | 0/10 | Toxic |
| | 150 | 0/10 | Toxic |
| | 100[a] | 10/10 | 0.8, 0.8[b] |
| | 50 | 10/10 | 0.8 |
| | 25 | 10/10 | 0 |
| TCNU | 50 | 0/5 | Toxic |
| | 30[a] | 10/10 | 11.6, 12.4[b] |
| Chlorozotocin | 80 | 3/9 | Toxic |
| | 60[a] | 9/9 | 0, 0[b] |

[a]Maximum tolerated dose. [b]Two independent experiments.

100 mgkg$^{-1}$ip. The increases in median survival times were significant at the 1% level when compared to the control group using the Mann-Whitney U test. However, at the maximum tolerated doses the activity of Clomesone was inferior to that of TCNU and Chlorozotocin. Anti-tumour activity against the relatively nitrosourea-sensitive solid sc MAC 13 tumour is presented in Table III. Although the tumour weight inhibitions achieved by Clomesone at 100 mgkg$^{-1}$ip and 50 mgkg$^{-1}$ip or iv were significant ($P = 0.05$; student's $t$-test), they were less than that achieved by TCNU. Clomesone was inactive against the relatively nitrosourea-resistant solid sc MAC 26 tumour as described in Table IV. The relative tumour volumes of the group treated with the maximum tolerated dose of 100 mgkg$^{-1}$ip were not significantly different ($P > 0.1$) from those of the control group when analysed using the student's $t$-test.

The spleen colony forming unit assay calibration curve is shown in Figure 2. Linear regression analysis showed a strong positive correlation ($r = 0.997$) between the number of surface spleen colonies and the number of marrow cells inoculated. Linearity was observed over the range $1.2 \times 10^4$–$7.4 \times 10^5$ cells injected. A cell inoculum below $1.2 \times 10^4$ produced no visible surface colonies while a cell inoculum of $3.4 \times 10^6$ resulted in the total repopulation of the spleen with no discrete colony formation.

Acute bone marrow toxicity following ip drug administration is described in Table V. The results at the maximum tolerated dose for each drug represent the survival fractions obtained in two independent experiments. Clomesone was less myelosuppressive than TCNU and Chlorozotocin but still produced a 5-fold reduction in colony forming units at the maximum tolerated dose of 100 mgkg$^{-1}$.

The solvent extraction procedure used in the pharmacokinetic studies gave recoveries for six replicate plasma samples, at a concentration of 1 $\mu$gml$^{-1}$, of 98.8% for Clomesone and 88.9% for the internal standard, 2-chloroethyl-p-toluenesulphonate with an overall intersample variation for the assay of 9.4%. Adequate separation of Clomesone and the internal standard from plasma interferents was achieved under the assay operating conditions and a typical chromatogram is presented in Figure 3(a). No underlying plasma interferent peaks co-eluted with Clomesone as shown in Figure 3(b). The limit of detection for Clomesone in plasma was 0.05 $\mu$gml$^{-1}$. A drug calibration curve was constructed using least squares linear regression analysis and linearity was observed in the concentration range 0.05–10 $\mu$g ml$^{-1}$ ($r = 0.987$).

The plasma concentration of Clomesone following ip administration of 50 mgkg$^{-1}$ as a function of time is shown in Figure 4. Each point is the mean of duplicate samples and each curve represents an independent experiment. Peak plasma levels were reached within 2 min of drug administration and ranged from 18.1-35.9 $\mu$gml$^{-1}$ with a mean value of 28.0 $\mu$gml$^{-1}$ while the mean terminal half-life was 8.1 min (range 4.8-10.8 min). The areas under the plasma concentration versus time curves ranged from 5.70–8.04 $\mu$ghml$^{-1}$ with a mean value of 6.77 $\mu$ghml$^{-1}$. Clomesone degradation in tissue culture medium exhibited 1st order kinetics with a rate constant (k) of 0.205 and a $t_{1/2}$ of 3.38 h. The c × t products, shown in Table I, corresponding to the initial drug concentrations required to achieve a 50% cell kill *in vitro* ranged from 54– > 528 $\mu$ghml$^{-1}$.

**Figure 2** Spleen colony forming unit assay calibration curve (points are mean ± SD; $n = 6$).

**Table V** Bone marrow toxicity following ip administration

| Compound | Dose ($mg\,kg^{-1}$) | No. colonies observed (mean ± SD; n = 6) | Survival fraction |
|---|---|---|---|
| Clomesone | 100[a] | 4.8 ± 0.75 | 0.20 |
| | 100[a] | 6.5 ± 2.0 | 0.16 |
| | 50 | 11.8 ± 1.17 | 0.59 |
| | 25 | 17.8 ± 0.84 | 0.80 |
| TCNU | 30[a] | 0 | 0 |
| | 30[a] | 0 | 0 |
| | 10 | 6.0 ± 0.9 | 0.20 |
| | 5 | 14.0 ± 1.2 | 0.61 |
| Chlorozotocin | 60[a] | 0.33 ± 0.52 | 0.01 |
| | 60[a] | 2.25 ± 0.50 | 0.05 |
| | 30 | 21.4 ± 1.14 | 0.80 |
| | 15 | 24.3 ± 0.96 | 0.86 |

[a]Maximum tolerated dose, two independent experiments.

### Discussion

Examination of the *in vitro* activity of Clomesone in a panel of established murine and human tumour cell lines revealed that, in general, the human cell lines were insensitive. The colon adenocarcinoma HCLO and the leukaemia K562 were the only human cell lines that were sensitive at similar $IC_{50}$ values. Similarly, in the murine cell lines the concentration required for a 50% cell kill of the line derived from the relatively nitrosourea-resistant MAC 26 tumour was of the

Figure 3 a, Chromatogram of mouse plasma extract containing Clomesone and internal standard (1 = Clomesone; 2 = internal standard). b, Chromatogram of mouse plasma extract containing internal standard only (1 = position of Clomesone peak; 2 = internal standard).

Figure 4 Plasma concentration of Clomesone following administration of 50 mg kg$^{-1}$ip (points are mean values; $n = 2$; three independent experiments).

same order of magnitude as that required by the leukaemia WEHI-3B. These findings suggested that Clomesone had no preferential specificity for cell lines derived from solid tumours.

The lack of selectivity was confirmed by the results of the *in vivo* anti-tumour activity and toxicity studies. The activity of Clomesone in the MAC tumour system *in vivo* was not impressive. Moderate activity was exhibited against the ascitic MAC 15A. The solid sc MAC 13 did respond to Clomesone over a broad dose range but the maximum anti-tumour activity exhibited was not great in this relatively nitrosourea-sensitive tumour. No significant activity was demonstrated against the relatively nitrosourea-resistant solid sc MAC 26 tumour. Thus, the anti-tumour activity of Clomesone was similar, if slightly inferior, to that of the non-carbamoylating nitrosourea, Chlorozotocin, and it was much less effective than the carbamoylating nitrosourea, TCNU, which remains the most active nitrosourea analogue in the MAC tumour system to date. Although Clomesone exhibited less bone marrow toxicity compared to both TCNU and Chlorozotocin, the reduction in colony forming units produced at the maximum tolerated dose was still marked. In addition, the dose of TCNU which produced a similar level of bone marrow toxicity has been reported to give a better response than Clomesone against the nitrosourea-sensitive MAC 13 tumour (Bibby *et al.*, 1988*a*).

The development of the selective and sensitive method for the quantitative determination of Clomesone in mouse plasma allowed the pharmacokinetic behaviour of the drug to be characterised with a view to relating the activity-toxicity pattern to bioavailability. The AUC values for Clomesone following a single ip injection of 50 mg kg$^{-1}$, when considered in conjunction with the *in vitro* c × t product for MAC 26, suggested that the inactivity *in vivo* was due to ineffective anti-neoplastic drug concentrations at the tumour site. A higher drug exposure of the tumour cells could be achieved *in vitro* as the drug half-life in tissue culture medium of 3.38 h reported in this study was much longer than the previously reported $t_{1/2}$ of Clomesone in mouse plasma of 0.85 h (Chan & Barrientos, 1988). The dose of 50 mg kg$^{-1}$ was chosen as the optimal dose as the higher maximum tolerated dose of 100 mg kg$^{-1}$ resulted in no improvement in anti-tumour activity at the expense of increased bone marrow toxicity.

Clomesone was selected for clinical trial on the basis that although it was only as effective as the chloroethylnitrosoureas in some murine tumour models, its chemistry suggested that it would be more toxicologically selective. The broader pre-clinical evaluation performed in this study could find no evidence to support this theory. On the contrary, the findings suggested that the effectiveness of Clomesone, in common with the chloroethylnitrosoureas, would be limited by myelosuppression. Whether the results of this study will be reflected in the clinical setting remains to be seen and the results of the recently completed Phase I clinical trial are awaited with great interest.

This work was supported by the Association for International Cancer Research, Pharmacia Leo Therapeutics, Helsingborg, Sweden, the Whyte Watson/Turner Cancer Research Trust and Bradford's War on Cancer, Bradford, West Yorkshire, UK. The authors would also like to thank the CRC for donating the gas chromatograph with the electron capture detector.

### References

BIBBY, M.C., DOUBLE, J.A. & MORRIS, C.M. (1988*a*). Anti-tumour activity of TCNU in a panel of transplantable murine colon tumours. *Eur. J. Cancer Clin. Oncol.*, **24**, 1361–1364.

BIBBY, M.C., DOUBLE, J.A., WAHED, I.A., HIRBAWI(ABU-KHALAF), N. & BAKER, T.G. (1988*b*). The logistics of broader pre-clinical evaluation of potential anti-cancer agents with reference to anti-tumour activity and toxicity of mitozolomide. *Br. J. Cancer*, **58**, 139–143.

BOYD, M.R. (1989). Status of the NCI preclinical antitumor drug discovery screen. In *Cancer: Principles and Practice of Oncology Updates*, De Vita, V.T. Jr, Hellman, S. & Rosenberg, S.A. (eds), Lippincott: Philadelphia, **3**, 1–12.

CHAN, K.K. & BARRIENTOS, A. (1988). Analysis of Clomesone in plasma by gas chromatography-electrolytic conductivity detection. *J. Chromatography*, **428**, 331–339.

CHENG, C.J., FUJIMURA, S., GRUNBERGER, D. & WEINSTEIN, I.B. (1972). Interaction of 1-(2-chloroethyl)-3-cyclohexyl-1-nitrosourea (NSC 79037) with nucleic acids and proteins *in vivo* and *in vitro*. *Cancer Res.*, **32**, 22–27.

CORBETT, T.H., VALERIOTE, F.A. & BAKER, L.H. (1987). Is the P388 murine tumor no longer adequate as a drug discovery model? *Invest. New Drugs*, **5**, 3–20.

DOUBLE, J.A. & BALL, C.R. (1975). Chemotherapy of transplantable adenocarcinomas of the colon in mice. *Cancer Chemother. Rep.*, **59**, 1083–1089.

DOUBLE, J.A. BALL, C.R. & COWEN, P.N. (1975). Transplantation of adenocarcincomas of the colon in mice. *J. Natl Cancer Inst.*, **54**, 271–275.

DOUBLE, J.A. & BIBBY, M.C. (1989). Therapeutic index: a vital component in selection of anticancer agents for clinical trial. *J. Natl Cancer Inst.*, **81**, 988–994.

DYKES, D.J., WAUD, W.R., HARRISON, S.D. Jr, LASTER, W.R. Jr, GRISWOLD, D.P. Jr, SHEALY, Y.F. & MONTGOMERY, J.A. (1989). Anti-tumor activity of 2-chloroethyl(methylsulfonyl)methanesulfonate (Clomesone, NSC 338947) against selected tumor systems in mice. *Cancer Res.*, **49**, 1182–1186.

ERICKSON, L.C., BRADLEY, M.O., DUCORE, J.M., EWIG, R.A.G. & KOHN, K.W. (1980). DNA crosslinking and cytotoxicity in normal and transformed human cells treated with antitumor nitrosoureas. *Proc. Natl Acad. Sci. USA*, **77**, 467–471.

GERAN, R.I., GREENBERG, N.H., MACDONALD, M.M., SCHUMACHER, A.M. & ABBOTT, B.J. (1972). Protocols for screening chemical agents and natural products against animal tumors and other biological systems (third edition). *Cancer Chemother. Rep.*, **3**, 1–103.

GIBSON, N.W., ERICKSON, L.C. & KOHN, K.W. (1985). DNA damage and differential cytotoxicity produced in human cells by 2-chloroethyl(methylsulfonyl)methanesulfonate (NSC338947), a new DNA-chloroethylating agent. *Cancer Res.*, **45**, 1674–1679.

GIBSON, N.W., HARTLEY, J.A., STRONG, J.M. & KOHN, K.W. (1986). 2-Chloroethyl(methylsulfonyl)methanesulfonate (NSC-338947), a more selective DNA alkylating agent than the chloroethylnitrosoureas. *Cancer Res.*, **46**, 553–557.

LOWN, J.W., MCLAUGHLIN, L.W. & CHANG, Y.-M. (1978). Mechanisms of action of 2-haloethylnitrosoureas on DNA and its relation to their antileukemic properties. *Bioorg. Chem.*, **7**, 97–110.

LUNN, J.M., CARMICHAEL, J., BIBBY, M.C., DOUBLE, J.A. & HARRIS, A.L. (1989). O$^6$-alkylguanine-DNA alkyltransferase expression and glutathione transferase action in MAC tumours correlate with intrinsic resistance to nitrosoureas and chlorambucil *in vivo*. *Br. J. Cancer*, **60**, 498.

MARSONI, S., HOTH, D., SIMON, R., LEYLAND-JONES, B., DE ROSA, M. & WITTES, R.E. (1987). Clinical drug development: an analysis of Phase II trials, 1970–1985. *Cancer Treat. Rep.*, **71**, 71–80.

MCELHINNEY, R.S., MCCORMICK, J.E., BIBBY, M.C., DOUBLE, J.A., ATASSI, G., DUMONT, P., PRATESI, G. & RADACIC, M. (1989). Nucleoside analogues: 8. Some isomers of B.3839, the original 5-fluorouracil/nitrosourea molecular combination, and their effects on colon, breast and lung tumours in mice, *Anti-Cancer Drug Design*, **4**, 1–20.

PELFRENE, A., MIRVISH, S.S. & GOLD, B. (1976). Brief communication: induction of malignant bone tumors in rats by 1-(2-hydroxyethyl)-1-nitrosourea. *J. Natl Cancer Inst.*, **56**, 445–446.

PHILLIPS, R.M., BIBBY, M.C. & DOUBLE, J.A. (1990). A critical appraisal of the predictive value of *in vitro* chemosensitivity assays. *J. Natl Cancer Inst.*, **82**, 1457–1468.

PHILLIPS, R.M., HULBERT, P.B., BIBBY, M.C., SLEIGH, N.R. & DOUBLE, J.A. (1992). *In vitro* activity of the novel indoloquinone EO-9 and the influence of pH on cytotoxicity. *Br. J. Cancer*, **65**, 359–364.

SARIBAN, E., ERICKSON, L.C. & KOHN, K.W. (1984). Effects of carbamoylation on cell survival and DNA repair in normal human embryo cells (IMR-90) treated with various 1-(2-chloroethyl)-1-nitrosoureas. *Cancer Res.*, **44**, 1352–1357.

SCHOFIELD, R. (1986). Assessment of cytotoxic injury to bone marrow. *Br. J. Cancer*, **53**, Suppl. VII, 115–125.

SHEALY, Y.F., KRAUTH, C.A. & LASTER, W.R. Jr (1984). 2-Chloroethyl(methylsulfonyl)methanesulfonate and related (methylsulfonyl)methanesulfonates. Antineoplastic activity *in vivo*. *J. Med. Chem.*, **27**, 664–670.

SHEALY, Y.F., KRAUTH, C.A., STRUCK, R.F. & MONTGOMERY, J.A. (1983). 2-Haloethylating agents for cancer chemotherapy. 2-Haloethyl sulfonates. *J. Med. Chem.*, **26**, 1168-1173.

SWENSON, D.H., FREI, J.V. & LAWLEY, P.D. (1979). Synthesis of 1-(2-hydroxyethyl)-1-nitrosourea and comparison of its carcinogenicity with that of 1-ethyl-1-nitrosourea. *J. Natl Cancer Inst.*, **63**, 1469–1473.

TILL, J.E. & MCCULLOCH, E.A. (1961). A direct measurement of the radiation sensitivity of normal mouse bone marrow cells. *Rad. Res.*, **14**, 213–222.

TONG, W.P., KOHN, K.W. & LUDLUM, D.B. (1982). Modifications of DNA by different haloethylnitrosoureas. *Cancer Res.*, **42**, 4460–4464.

TWENTYMAN, P.R. & LUSCOMBE, M. (1987). A study of some variables in a tetrazolium dye (MTT) based assay for cell growth and chemosensitivity. *Br. J. Cancer*, **56**, 279–285.